# A Systematic Review of the Use of Commercial Wearable Activity Trackers for Monitoring Recovery in Individuals Undergoing Total Hip Replacement Surgery

**DOI:** 10.34133/2022/9794641

**Published:** 2022-10-26

**Authors:** Nasibeh Babaei, Negin Hannani, Nader Jafarnia Dabanloo, Shayan Bahadori

**Affiliations:** ^1^Department of Biomedical Engineering, Science And Research Branch, Islamic Azad University, Tehran, Iran; ^2^Faculty of Health and Social Science, Bournemouth University, Bournemouth, UK

## Abstract

The innovation of wearable devices is advancing rapidly. Activity monitors can be used to improve the total hip replacement (THR) patients' recovery process and reduce costs. This systematic review assessed the body-worn accelerometers used in studies to enhance the rehabilitation process and monitor THR patients. Electronic databases such as Cochrane Database of Systematic Reviews library, CINAHL CompleteVR, Science Citation Index, and MedlineVR from January 2000 to January 2022 were searched. Due to inclusion criteria, fourteen eligible studies that utilised commercial wearable technology to monitor physical activity both before and after THR were identified. Their evidence quality was assessed with RoB 2.0 and ROBINS-I. This study demonstrates that wearable device technology might be feasible to predict, monitor, and detect physical activity following THR. They could be used as a motivational tool to increase patients' mobility and enhance the recovery process. Also, wearable activity monitors could provide a better insight into the individual's activity level in contrast to subjective self-reported questionnaires. However, they have some limitations, and further evidence is needed to establish this technology as the primary device in THR rehabilitation.

## 1. Introduction

Over the last decades, the number of individuals undergoing total hip replacement (THR) surgery has increased exponentially [1]. Rehabilitation services are a key component to continuing treatment for THR patients, although more efforts are still needed to identify the optimal approach for recovery [2].

Patients' function is usually assessed by patient-reported outcome measures (PROMs) and clinical visits [3]. Nevertheless, it has been shown that PROMs used to monitor patients' health aspects do not always provide the most reliable and accurate insight on recovery and activity level [4]. Since self-reported physical activity mostly indicates bias and overestimation, and patients are sometimes unable to explain the exact recovery process [5]. In addition, clinical visits and face-to-face physical therapy have some drawbacks like cost and unavailability, especially for people living in rural and remote areas [6].

In order to improve pain and physical function, remote rehabilitation could be efficient and comparable to standard outpatient rehabilitation [7]. A study by Austin et al. [8] showed no difference between outcomes of unsupervised home-based therapy and formal physiotherapy. More interestingly, the cost of rehabilitation in the unsupervised home exercise group was lower.

Wearable activity monitors can provide an alternative to standard rehabilitation by encouraging self-management. Initially, the number of connected wearable devices worldwide was 325 million in 2016 and will have increased substantially to above 1000 million by 2022 [9]. Thanks to powerful microchips and sensors, wearable devices are able to measure practical data (biometric information). Despite some drawbacks regarding user-friendliness, cost, and comfort, they have been used in different medical fields to monitor cardiology [10], seizures [11], systematic disorders [12], COVID-19 [13], cancer [14], TKR [15], and spinal surgery [16]. Moreover, with the advent of commercial activity monitors, monitoring patients before and after surgery have become more accurate [5].

Several studies have assessed the feasibility and reliability of wearable devices in different aspects of recovery in individuals undergoing THR surgery. Findings highlight some limitations of current commercial activity monitors, such as lack of accuracy at slow walking speeds [17, 18]. Furthermore, there are discrepancies in objective data measured on physical activity (PA) and sleep in contrast to PROMs' scores [19–21]. Moreover, some studies have used these activity monitors to gain valuable data to assist in the optimisation of hip wear simulator studies [22]. In terms of sleep parameters, activity monitors are poor at detecting sleep, so if the patient is lying motionless but fully awake, the tracker records it as sleep [23]. Since our last review on this topic [24], there has been a surge of publications using commercial activity monitors for monitoring THR patients. Therefore, this review aims to systematically identify all studies that utilised commercial activity monitors to measure activity in individuals both before and after THR and further debates the application of these activity monitors as a rehabilitation intervention.

## 2. Method

This review is reported according to the Preferred Reporting Items for Systematic Reviews and Meta-Analyses (PRISMA) statement [25]. A computer-based search was completed in February 2022 using the mySearch Database (Bournemouth University). This included the Cochrane Database of Systematic Reviews library, CINAHL CompleteVR, Science Citation Index, and MedlineVR. Articles published in English from January 2000 to January 2022 were reviewed. Search strategy terms are outlined in Table 1.

Duplicates were removed after completing the database search. SB screened titles and abstracts of studies, then the full-text documents were reviewed, and studies that met inclusion/exclusion criteria were gathered. Also, the references of the selected articles were screened by NH and NB, and other articles meeting inclusion criteria were also included.

The Health Research Organization (HRA) Ethics Database [26] confirmed that ethics approval was not required since this study used publicly available information and did not interact with patients or obtain personally identifiable information.

### 2.1. Data Extraction

In order to form the summary table (Table 2), information was gathered on the study population, its aim and outcomes, and the type of device utilised.

### 2.2. Risk of Bias

RoB 2.0 tool [27] and ROBINS-I tool [28] were used to assess the risk of bias in each randomised and nonrandomised study, respectively. RoB 2.0 consists of domains including the randomisation process, deviations from intended interventions, missing outcome data, measurement of the outcome, and selection of the reported result. The answer options for an overall RoB 2.0 judgment are as follows: “low risk”, “high risk”, and “some concerns”. ROBINS-I is structured into seven domains: confounding, selection of participants into the study, classification of interventions, deviations from intended interventions, missing data, measurement of outcomes, and selection of the reported result for reaching the risk of bias judgments. The answer options for an overall ROBINS-I judgment are as follows: “low risk”, “moderate risk”, “serious risk”, “critical risk”, and “No information”. Two reviewers (NB and NH) assessed each paper independently. Any disagreements between reviewers were discussed with SB and resolved by consensus.

## 3. Result

### 3.1. Selection Process

**Figure 1:**
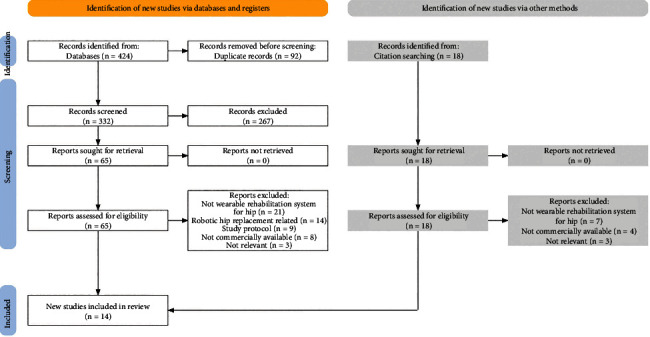
Database search records.

Four hundred twenty-four records were found through database searching (Figure 1). After removing duplicates, the titles and abstracts of the 322 records were screened. Sixty-five full-text studies were reviewed. Then, records were excluded if they were not wearable rehabilitation system for hip (*n* = 21), only focused on robotic hip replacement operation (*n* = 14), were study protocol (*n* = 9), were not commercially available (*n* = 8), and were not relevant (*n* = 3). Moreover, an additional four records were identified from citation searching. Consequently, 14 studies were eligible that met the eligibility criteria.

### 3.2. Participants

A total of 2487 participants, of which about 70% were female, took part in these fourteen studies. The most common inclusion criteria were participants aged 61 years old and over, could walk more than 5 kilometres a day, speak English, understand instructions and were able to complete surveys, and underwent primary THR due to OA.

Generally, according to the studies' requirements, demographic data, type of surgery, preoperative information, narcotic usage, length of stay, and assistive device usage were recorded from medical records or self-reported data.

### 3.3. Devices

Commercial wearable activity monitors such as Sportline, Lifecorder, New Lifestyles NL1000, Fitbit, Garmin VivofitR2, Nokia Go, FitPro, Fitbit Zip, Apple watch, and Letscom were used in these studies. These activity monitors monitored step count and physical activity using an accelerometer sensor. Mio Activity Tracker, Fitbit Flex, Garmin VivofitR2, and Apple watch were equipped with optical heart rate; therefore, they not only tracked physical activity but also measured heart rate and sleeping time. Lifecoder EX and Lumo run were worn on a belt at the waist level of the sacrum, whereas other pedometers were worn on the wrist. Data were either uploaded via Bluetooth or submitted by the patients. In several studies, the wearable activity monitors' data was claimed to be reliable and valid for the assessment of step count due to previous documents [29–33].

### 3.4. Interventions

All studies used wearable activity monitors to monitor patients and obtain data, including the number of steps, number of sleeping hours, heart rate, caloric expenditure, and physical activity. Various methods were utilised based on the study's aim and objective. A number of studies combined mobile-health applications like the MoveUp or Mymobility platform with activity monitors [31, 34]. In several studies, the patients were divided into control and intervention groups. The control groups mostly received the usual care as prescribed by the surgeon consisting of physiotherapy pre- and postsurgery and a single session in which they received information about the operation, walking with crutches, and exercises that would be performed in the postoperative phase [34–36]. The intervention groups received different interventions, including supervised sessions and training based on the patient's individual goals (a variety of vocational and recreational activities such as basketball, golf, jogging, and curling) [35], 30-minute walk per day [36], educational materials pre- and postoperatively [34], and home-based exercise program [34–36]. The home-based exercise programme involved six to eight exercises, performed three times per day, six days per week for six weeks postoperatively [34], training according to the principles of functional task exercise developed by Oosting et. al and de Vreede et al. [36–38], and exercises such as walking, climbing stairs, and rising out of a chair with symmetrical force and movement between the legs [35]. In one study, the feedback group received a daily step goal (7000 steps by week 6) and was allowed to see the number of steps, while the other group did not [29].

Lebleu et al. [31] prescribed personalised, daily exercises, and feedback through a tablet. Patients in the study of Goeb et al. [39] were instructed to avoid the “leg-shaving position” as a postoperative precaution. Studies ranged from approximately 4 to 390 days in duration, with an average length of 113 days.

### 3.5. Use of Wearable Activity Monitors

Studies found that the data from commercial wearable activity monitors will be beneficial in different aspects. Including predicting the recovery process, motivation to increase mobility [29, 40], remote monitoring [39, 40], increasing patients' performance [29, 31], providing personalised care [31], and eliminating the need for self-reported data [39]. Furthermore, wearable activity monitors may measure ambulation more reliably [40] and accurately [32] than self-reported assessments, and they also make it possible to train and track frail elderlies at home [36]. Daskivich et al. [41] also stated that ordering of ambulation, assessing, and monitoring mobility by activity monitors are more accurate and cost-effective. This technology can provide early intervention by stratification patients' risk in the postoperative period [42], as well as allow for early intervention in patients who are recovering slowly by detecting differences in levels of activity clearly [40].

Integrating the smartwatch data with a smartphone platform or electronic medical record could show real-time activity, reduce postoperative physiotherapy, and notify the patients and surgeons of the lack of progress [34, 41]. Also, the combination of activity monitors with machine learning can be used to predict PROMs [42]. Madara et al. [35] demonstrated that home-based exercise with a pedometer is feasible and useful.

Despite the information these devices could provide, Goeb et al. [39] found several technical errors that caused data to be recorded inaccurately.

### 3.6. Adherence and Satisfaction

Walt et al. [29] and Madara et al. [35] found that satisfaction with surgery outcomes was higher in patients using wearable activity monitors. Also, satisfaction with treatment and adherence to the training were observed in the study of Oosting et al. [36]. However, Tang et al. [43] stated that they could not find any differences in physical activity or sleep over time because of patients' lack of adherence. This nonadherence was probably due to biases of accelerometry-based research associated with the difficulty of enforcing activity monitors wear throughout the study period, as reported in the study of Trost et al. [44]

### 3.7. Predicting Data

Personalised Activity Intelligence calculated by Mio Activity Tracker was identified to be an important feature in predicting total joint replacement (TJR) outcomes [42]. Preoperative step count could predict the three-month physical activity level, duration of crutches use, and preoperative symptoms [31]. Moreover, to categorise which data set is more likely to predict which PROM, Bini et al. [42] introduced the concept of distinguishing between quantitative and qualitative features of PROMs.

### 3.8. Physical Activity

The majority of the studies demonstrated an overall change in the number of daily steps and patient activity, which increased during the follow-up. Operation type [32, 40, 41], discharging to home or a nursing facility [40], age, BMI, and systemic disease [45] correlated with the activity level. Goeb et al. [39] found an improvement in pain for every 1000 steps walked on average. BMI greater or less than 30 kg/m2 did not show any differences in the level of activity in the study of Toogood et al. [40]. Comparing the two groups, the treatment group had a considerably higher mean daily step count [29] and used less postoperative physiotherapy [34]. There were no considerable differences in readmissions, complications, or outpatient visits among groups [39]. By the fourth week, 74% and 76% returned to work and driving. 26% used assistive devices, and 23% took pain medication six weeks postoperatively [39]. In the study of Lebleu et al. [31], patients reached their preoperative physical activity level at week 7, with no significant additional improvement by three months postsurgery. It is also reported that patients returned to near baseline levels over roughly three months [32, 43]. 35% of the variability of step count at three months could be explained by the number of days using crutches [31]. More activity before surgery was associated with better rehabilitation after surgery [32]. Fujita et al. [30] found no improvement in vigorous physical activity, although light and moderate physical activity improved one year after THR.

Goeb et al. [39] declared that modified postoperative precautions result in more freedom and activity level than traditional ones without increasing the risk of instability events. In this study, the step counts rose from 1098 at week 1 to 6069 at week 6. Also, Franklin [45] found that young patients with primary THR may not be as active as thought. In addition, Daskivich et al. [41] claimed that significantly lower odds of prolonged length of stay were associated with a higher step count of up to 1000 steps on the first day after surgery. Therefore, a 1000-step daily goal was suggested for ambulation in the early postoperative period after the major surgery. In the Walt's study [29], the weekly step goals were selected based on the mean daily steps observed in the study of Twiggs et al. [46]. The goal of 7000 steps by week six was selected as this is the recommended daily step count for healthy older adults [47].

Some studies measured the steps based on the distance travelled [33, 36, 43, 45]. The average daily steps in these studies were 4464 and 3562 per day pre- and postsurgery, respectively [33, 36, 43].

### 3.9. Physical Performance Measurements

The intervention group showed more remarkable improvement in the six-minute walk test (6MWT), hip abduction strength on the nonsurgical side, and force symmetry during sit-to-stand than the control group [35]. There were no significant differences in mean hip flexion, single leg stance (SLS) and timed up and go (TUG) between the two groups [34].

### 3.10. Patient-Reported Outcome Measures

Different kinds of questionnaires were completed by patients as required. Although several studies stated that there might be a lack of correlation between PROMs and activity monitor data [42, 43, 45], a number of studies found a correlation [30, 39]. Also, Bini et al. [42] determined that qualitative data were associated with PROMs such as the Hip Disability and Osteoarthritis Outcome Score (HOOS) and Knee Osteoarthritis Outcome Score (KOOS) surveys. At the same time, quantitative sensor data were more likely to correlate with functional outcomes as measured by the Veterans RAND 12-item Health Survey (VR-12). Moreover, not all questionnaires are appropriate for each individual [36]. Madara et al. [35] declared that the intervention group had more improvement in HOOS scores than the control group.

### 3.11. Sleep

Sleep data may not correlate with patient-reported outcomes in early follow-ups after THR [43]. While patients report improvements in subjective clinical outcomes, the pedometer's findings suggest a return to preoperative levels in objective measures at three months [43]. The average daily sleeping time was reported as 368 minutes preoperatively, 312 minutes at early postoperative, and 633 minutes at three months postoperatively [43]. During 12 weeks postsurgery, Karas et al. [32] reported a decrease in sleep efficiency.

Activity and heart rate data were generally observed to be less variable than sleep data [32], possibly due to poorer nighttime data coverage and the relatively low accuracy of current models for estimating sleep metrics from consumer wearables [48].

### 3.12. Cup Wear Rate

Bennet et al. [33] and Franklin [45] found no correlation between pedometer activity data and wear rate. However, Bennet et al. [33] demonstrated that patients with the lowest wear rates showed a strong positive relationship between activity level, wear rate and multidirectional motion, and the sliding distance.

### 3.13. Risk of Bias in Individual Studies

The quality of the evidence was examined with the risk of bias tool in these fourteen studies (Table 3). In ROBINs tool, risk of bias in 6 studies was judged to be serious in at least one domain but not critical in any domain, and 4 studies were moderate for all domains. In RoB, 1 study was at high risk of bias, and 3 studies raised some concerns in at least one domain for this result but were not at high risk of bias for any domain.

## 4. Discussion

Wearable physical activity monitors have the ability to enhance the recovery process by providing physical activity information related to steps, sleep, heart rate, and energy expenditure. In addition to helping patients, wearables can provide valuable information for surgeons and physiotherapists. Activity monitors have been utilised in different areas of healthcare research. It is worth noting that efforts are still needed to prove the ability of this technology to be the main device in THR care. Our previous study conducted a review of commercial wearable activities to monitor patients following THR. Nevertheless, recently, there has been an increase in documents in this field of research. Hence, the aim of this study is to summarise recent findings on commercial body-worn activity monitors in THR patients' rehabilitation.

This systematic review found that, overall, fourteen studies utilised body-worn accelerometers for THR patients. The majority of included studies were not randomised, and sample sizes were often small. Patients and outcome assessors were not blinded in most of the studies. In some studies, there is a possibility of data inaccuracies as the patients recorded the number of steps on the questionnaire. The bias levels were assessed by RoB 2.0 and ROBINS-I (Table 3).

Patient-reported outcomes' measures are a common way to assess the improvement of quality of care. However, it was reported that subjective measurements could not be as reliable as objective recordings [40]. Several studies found little correlation between the pedometer measurements and PROMs [42, 43, 45]. For instance, in some cases, patients reported improvement in PROMs, while no improvement was seen in activity monitors' recordings [43]. Harding et al. [49] found that personal beliefs about the physical activity may explain the discrepancy between subjective and objective outcomes. Because it satisfies patients to know that they can now be active if they want, patients overestimate their activity level. Wearable activity monitors seem to be a more reliable and accurate tool for physicians and researchers to estimate physical activity than PROMs.

Some included studies demonstrated that activity monitors could be one of the features that help to predict the rehabilitation process [30–32, 39, 42]. It can also be combined with machine learning to predict PROMs postoperatively [42]. The studies mentioned some benefits of prediction that can be provided for patients and surgeons. For example, postoperatively predicting the activity level can help surgeons prescribe personalised care [31] and earlier intervention for patients who may recover slowly to prevent poor outcomes [50]. Moreover, it can provide the patients with realistic expectations of their activity after the surgery [30]. McDonald et al. [51] found that awareness of recovery leads to faster mobilisation of patients after surgery as this information relieves anxiety and empowers them to participate in their recovery actively.

Remote patient monitoring can be feasible using wearable activity monitors [39, 40]. In favour of remote monitoring, saving health care resources, reducing unnecessary medical care, eliminating readmission, and decreasing costs might be possible. Moreover, the combination of wearable device technology with mobile applications provides opportunities to prescribe home-based exercises and monitor the completion and accuracy [34, 35]. Some evidence has shown that physiotherapy rehabilitation exercises, whether unsupervised at home or supervised by a physiotherapist, appear to be equally effective without any difference in pain or function [52, 53]. Therefore, unsupervised exercise using activity monitors and mobile applications could be replaced safely with physiotherapy visits and would be a strategic way to reduce the financial burden.

Despite the benefits that pedometers may have in improving the rehabilitation process, there are still some limitations that may result in inaccurate data. Activity monitors, which use an accelerometer to detect steps, might not be capable of tracking steps while using assistive devices such as crutches and walkers [17, 18]. Therefore, several studies reported that ankle-based activity monitors could be more accurate than wrist-based devices [43, 54]. In addition, to detect sleep, an accelerometer sensor would not be accurate enough as it cannot distinguish between sleeping and lying down [23]. In this case, an optical heart rate sensor might be more reliable for assessing sleep. Technical issues such as errors in pairing with a phone or missing data can prevent accurate data. Some of these activity monitors are uncomfortable to wear with several studies showing that patients avoided wearing them and dropped out of the study [30, 39].

In order to compare devices, a summary of their different characteristics, such as sensor type, price, battery life, and tracking features, is presented in Table 4.

### 4.1. Future Research

Commercial wearable technologies are getting smarter, lighter, and more convenient wearables, although they still need to be developed and reach the perfect model.

THR patients need to wear the device for a long time, so ergonomics and battery life should be taken into account. Battery life is variable depending on the activity monitors, and it is not able to monitor patients during charging. Regarding ergonomics, it is important that the device does not get too hot, does not cause the body's reaction in contact with skin, and is convenient to wear. Also, the privacy and security of wearable devices are a concern because user information may be misused. Besides, Germany provides a good example of how to manage population health data, recognising strong privacy concerns [55]. In general, solutions should be provided concerning these issues.

Standards are required to be provided for researchers to compare results between wearable technologies. For step count [56] and sleep validity [57], the Consumer Technology Association (CTA) has established validation criteria and protocols. More standards and protocols should be developed to include energy consumption and free-living conditions.

In future research, the potential of continuous or high-frequency digital measures for clinical decision-making in personalised care should be assessed. Patients' characteristics should be considered in the clinical trial as important factors influencing the level of activity, such as occupation, place of residence, preoperative diagnoses, age, or BMI. Also, for a better comparison, larger sample size and the participation of both sexes are necessary.

Current studies are focused on step count as their measure of physical activity. However, it is worth noting that a recent study [58] highlighted the willingness of THR patients to utilise the Global Positioning System (GPS) technology, which may be a better option for 2 recording outdoor activity among this cohort.

Moreover, to find the most appropriate activity monitor for THR patients, further comparison of the performance among the sensors is still needed. In addition, combining artificial intelligence with commercial wearable activity monitors could widely help predict clinical outcomes [42], and more studies are needed for further investigation in this field. In order to avoid errors in activity monitoring, it is recommended that patients use simple activity monitors with user-friendly interfaces and do not record device data on the daily log by themselves [39]. Also, one way to minimise the biases in studies is to focus on methodological aspects, as it is impossible to blind carers or people delivering the interventions and participants in most studies.

### 4.2. Limitation

There are also limitations to this study that require acknowledgement. In this systematic review, only studies using commercial activity monitors were included, since these wearable activity monitors are growing rapidly in popularity both among general population and clinical researchers. Thus, we believe they stand in a strong position to establish themselves as a future device for clinical trials in contrast to more expensive and less user friendly research grade accelerometers. Moreover, despite our best effort, studies employed different protocols making generalisability difficult.

## 5. Conclusion

Commercial wearable activity monitors have the potential to play an essential part in increasing the level of activity in individuals' undergoing THR surgery, as well as offer health care providers objective assessments of their patients' daily activity patterns. Despite several drawbacks regarding the sleep detection and inaccuracy in step counting at low walking speeds, evidance suggests that wearable activity monitors could provide a better insight on the individual's activity level in contrast to subjective self-reported questionnaires. However, this review suggests that further evidence is needed to establish this technology as the primary device in THR rehabilitation.

## Figures and Tables

**Table 1:** Literature search strategy. MM (MeSh term).

Individual	(MM “arthroplasty, replacement, hip”) OR (MM “hip prosthesis”) (hip^∗^) N5 (arthroplast^∗^ OR prosthes^∗^ OR replace^∗^)

Wearable systems	AND Wearable^∗^ OR Tracker^∗^ OR Device^∗^ OR Sensor^∗^

Setting	AND Rehabilitation OR Recovery OR Therapy OR Treatment

“” used to find exact phrase. ^∗^ used to find all word with a common stem. N5 used to find all articles containing the keywords within five words.

**Table 2:** Wearable activity monitor article summary.

	Reference	Study population	Device technology	What does it do?	Wearability placement	Aim of study	Analysis	Outcomes of study
1	Franklin [45]	THR patients (*n* = 34)	Sportline digital pedometer (accelerometer)	Counts steps		To quantify the postoperative activity level in young patients after THR and to measure linear polyethylene wear rates	HHS, UCLA activity score, and preoperative patient demographics were recorded. Patients were randomly divided into 4 groups corresponding to the 4 seasons. Activity data consisted of a patient's average step length, and a daily record of mileage walked over 1 week. Linear wear rates were measured.	Increased BMI and age correlated with decreased gait cycles per year. Patients with the systemic disease were less active than patients with localized hip conditions. Walking activity as measured by a pedometer did not significantly correlate with in vivo wear rates. Obesity has been associated with decreased pedometer accuracy and may have influenced the quantification of gait cycles.

2	Bennett et al. [33]	THR patients (*n* = 153)	FitPro digital pedometer	Measures physical activity		To investigate the relationship between multidirectional motion at the hip joint and wear rate	Kinematic input from three-dimensional gait analysis was used to determine the wear paths. A subgroup of 100 patients wore the pedometer to collect activity data for a period Of 2 weeks. Annual cycles for each patient were determined by Extrapolating this data to 52 weeks. The relationship between annual sliding distance and wear rate was analyzed.	Poor correlations were found between aspect ratio and sliding distance and wear rate for the larger group and between annual sliding distance and wear rate for the subgroup.

3	Fujita et al. [30]	THR female patients (*n* = 62) Healthy control group (*n* = 38)	Kenz Lifecorder EX pedometer (accelerometer)	Counts steps and measures physical activity	Worn on a belt at waist level above the leg	To examine the changes in PA measured by a pedometer and in QoL in women undergoing THR	PA level (by a pedometer) and Qol (by short-form 8, OHS) were measured one-month pre-THR and 6 and 12 months post-THR in the intervention group, and the control group was measured once time. The outcome was compared with a control group.	Pedometer data will be useful for realistic expectations for recovery. The number of steps improved significantly. A contribution of PA to the improvement of QoL was seen. By 1-year post-THR, most of the PA levels became 80, and 90% of those in the control group and QoL scores became comparable between 2 groups.

4	Oosting et al. [36]	THR patients (*n* = 30) (15 intervention group/15 control group)	New lifestyles (NL1000) pedometer (accelerometer)	Measures physical activity		To investigate the feasibility and preliminary effectiveness of a home-based intensive exercise program to improve the physical health of frail elderly patients	The intervention group preoperatively trained functional activities and walking capacity twice a week for 3 to 6 weeks and received a pedometer. The control group received care as usual. Feasibility was determined on the basis of adherence to treatment, patient satisfaction, adverse events, walking distance, and intensity of exercise. Preliminary effectiveness was determined by self-reported and performance-based outcome measures of functions, activities, and participation at 2 to 4 days before admission, at discharge, and 6 weeks after discharge.	Intensive preoperative training at home is feasible for frail elderly patients. Patients were very satisfied. TUG test and 6MWT were different between groups preoperatively. No statistically significant changes were seen between the groups in the immediate postoperative period.

5	Toogood et al. [40]	THR patients (*n* = 33)	Fitbit (accelerometer)	Counts steps	Worn on the ankle on the operated side	To assess the feasibility and reliability of a remote mobility monitoring program and to examine objectively risk factors related to functional recovery	Patients were divided into subgroups based on age, BMI, surgical approach, and disposition to monitor activity using a tracker for 30 days postoperatively at home and in a nursing facility.	The wearable device was simple and easy to use, even for long periods. Increasing age, the use of the mini-posterior approach, and discharge to a nursing facility are factors associated with reduced early activity postoperatively. BMI had no influence. An objective measure of mobility may be more reliable than patients' subjective assessment.

6	Walt et al. [29]	TKR/THR patients (*n* = 163) (95 THR, 68 TKR)	The Garmin VivofitR2 (3-axis MEMS accelerometer)	Counts steps	Worn on wrist	To determine if feedback from a commercial activity monitor improves activity levels over the first 6 weeks of THR or TKR.	Participants were divided into two groups. The feedback group could see their daily step count (tThe other group could not) and were given a daily step goal (the other group was not). Daily step count and PROMs (KOOS and HOOS) were collected at 1, 2, 6 weeks, and 6 months postsurgery.	The feedback group had significantly higher activity levels. Commercial activity trackers may be a useful and effective adjunct after arthroplasty. There was no significant difference between the groups in PROMS.

7	Bini et al. [42]	THR, TKR patients (*n* = 22) (13 THR, 9 TKR)	Fitbit flex (accelerometer) Mio activity tracker (optical heart rate, 3-axis accelerometer) Lumo run (9-axis IMU, accelerometer, gyroscope, magnetometer, barometer)	Fitbit: Monitors daily activity (daily step counts, calories burned, distance walked, floors ascended and descended, number of minutes spent in each activity) Mio activity tracker: Monitors daily activity and heart rate (caloric expenditure, distance travelled, step counts, average resting heart rate, maximum daily heart rate) Lumo run: Tracks the pelvic motion (walking cadence, bounce (vertical oscillation), braking, pelvic rotation, sagittal pelvic tilt)	Fitbit: Worn on the wrist Mio activity tracker: Worn on the wrist Lumo run: Worn on either the belt or waistband of clothing at the level of the sacrum	To demonstrate the accuracy and feasibility of utilizing wearable sensors coupled with ML to predict PROMs.	The activity trackers collected 35 features from 4 weeks before to 6 weeks following surgery. HOOS, KOOS, and VR-12 were collected at both endpoints. Machine learning was used to identify features with the highest correlation with PROMs.	Data derived from commercial-grade wearable activity trackers can be used to predict patient-reported outcomes. No clear association was seen between preoperative activity levels with postoperative PROMs. ML can be used in combination with PGHD to predict 6-week PROM data as early as 11 days following TJR surgery.

8	Daskivich et al. [41]	Patients undergoing 8 major inpatient operations (*n* = 100) THR patients (*n* = 15)	Fitbit (accelerometer)	Counts steps	Worn on wrist	To define the distribution of digitally measured daily step counts, to assess the accuracy of physician assessment and ordering of ambulation, and quantify the association of digitally measured step count with the length of stay.	Sociodemographic and clinical data via review of the medical record were collected. Daily step count was passively monitored for the duration of hospitalization.	The study shows that activity monitors improve the accuracy of assessment of ambulation over the current standard of care. Higher step count up to 1000 steps on postoperative day 1 was associated with significantly lower odds Of prolonged length of stay, with no further decrease in odds after 1000 steps.

9	Lebleu et al. [31]	THR/TKR patients (*n* = 132) (66 THR, 66 TKR)	Nokia®go (accelerometer)	Counts steps	Worn on wrist	To determine perioperative factors that could help predict PA recovery.	Each subject received personalized and daily exercises and feedback through a tablet, and they were tracked from one week before until 3 months after surgery. PROM (HOOS, KOOS), the number of days of anti-inflammatory drugs intake, the number of days using crutches, and preoperative symptoms were recorded.	The PA level at 3 months could be moderately predicted by preoperative step count, duration of using crutches postsurgery, and preoperative symptoms level.

10	Madara et al. [35]	THR patients (*n* = 20) (10 control group/10 experimental group)	Fitbit zip ™ (accelerometer)	Tracks physical activity		To evaluate the feasibility of reducing supervised visits early after THR and retraining higher-level activities later in recovery.	The experimental group was prescribed a progressive home exercise program; this group's training was generally tailored to individual patient goals. The control group participated in usual rehabilitation care. Patients completed pPerformance-based functional tests, PROMs, satisfaction scores, and clinical measures. The results were compared between groups completed 2-4 weeks pre to 16 weeks postsurgery.	A progressive rehabilitation protocol that includes a period of home-based exercises followed by supervised movement training may benefit individuals after THR. This study had a positive effect on biomechanics and functional outcomes without compromising safety and the effect of the experimental. The intervention was most admirable for the 6MWT, nonsurgical hip strength, satisfaction, and movement symmetry.

11	Karas et al. [32]	Patients underwent surgery on a lower limb (*n* = 1,324) knee, or hip replacement (*n* = 196)	Fitbit flex (accelerometer, optical heart rate)	Captures steps, heart rate, and sleep data		To assess recovery relative to a personal baseline derived from long-term passive monitoring with consumer wearables	Achievement online platform was used to recruit participants and collect data on steps count, heart rate, and sleep derived by tracker up to 26 weeks before and after the surgery. Also, survey questions were answered.	Trajectories differ across surgery types. Leveraging long-term, passively collected wearable data promises to enable relative assessment of individual recovery.

12	Crawford et al. [34]	THR patients (*n* = 365) (198 control group, 167 treatment group with apple watch)	Apple watch (accelerometer, gyroscope, optical heart rate)	Tracks activity level	Worn on wrist	To assess the feasibility of the smartphone-based platform that can deliver noninferiority of clinical outcomes while reducing healthcare resource usage	Patients were randomized into 2 groups. The intervention group was provided with preoperative educational and exercise content along with postoperative educational material and an at-home app-based therapy programme for six weeks, while the control group received the usual care. PT use, THR complications, readmissions, and outpatient visits were evaluated, and outcomes between control and treatment groups were compared before surgery, at 30 days, and 90 days after surgery. Early outcomes were assessed, including HOOS, JR EQ-5D- 5 L, and SLS TUG.	Postoperative PT use was statistically lower in the smartphone-based care system group. However, there were no significant differences in complications, readmissions, outpatient visits, or the early outcomes between controls and treatment.

13	Goeb et al. [39]	THR patients (*n* = 82)	Letscom ID115Plus HR (accelerometer)	Counts steps	Worn on wrist	To assess the function of patients undergoing THR given modified precautions and to examine wrist-based activity trackers	Patients wore devices for 1 week preoperatively and 6 weeks postoperatively. Precautions included only the avoidance of the “leg-shaving” position. Patient progress in the early postoperative period was assessed with a rehab milestone questionnaire. Patients submitted step-counter data from the activity tracker.	Although the use of a wrist-based tracker was useful, several technical errors limit the ability of this wearable to record data accurately. A significant correlation was found between increased weekly steps and improved HOOS-JR scores after THR. Most patients had returned to work, resumed driving, and were no longer using assistive devices by the fourth week after surgery.

14	Tang et al. [43]	THR patients (*n* = 41)	Fitbit flex (accelerometer, optical heart rate)	Counts steps and measures sleeping time	Worn on wrist	To compare accelerometry-measured physical activity and sleep to patient-reported outcomes	HOOS-JR scores, outcome data of tracker, and sleeping medications were collected the day after their preadmission, 1 to 2 weeks, 1 month, and 3 months postoperatively.	Patients reported remarkable improvements in activity level and sleep, whereas accelerometry measurements did not correlate with that.

**Table 3:** Summary of risk of bias in included studies.

Reference	Tool	Risk of bias	Judgment across domains
Franklin [45]	ROBINS-I	Serious risk of bias	The study is judged to be at serious risk of bias in at least one domain but not at critical risk of bias in any domain.
Bennett et al. [33]	RoB 2.0	Some concerns	The study is judged to raise some concerns in at least one domain for this result but not to be at high risk of bias for any domain.
Fujita et al. [30]	ROBINS-I	Serious risk of bias	The study is judged to be at serious risk of bias in at least one domain but not at critical risk of bias in any domain.
Oosting et al. [36]	RoB 2.0	High risk of bias	The study is judged to have some concerns for multiple domains in a way that substantially lowers confidence in the result.
Toogood et al. [40]	ROBINS-I	Moderate risk of bias	The study is judged to be at low or moderate risk of bias for all domains.
Walt et al. [29]	RoB 2.0	Some concerns	The study is judged to raise some concerns in at least one domain for this result but not to be at high risk of bias for any domain.
Bini et al. [42]	ROBINS-I	Moderate risk of bias	The study is judged to be at moderate risk of bias.
Daskivich et al. [41]	ROBINS-I	Moderate risk of bias	The study is judged to be at low or moderate risk of bias for all domains.
Lebleu et al. [31]	ROBINS-I	Moderate risk of bias	The study is judged to be at low or moderate risk of bias for all domains.
Madara et al. [35]	ROBINS-I	Serious risk of bias	The study is judged to be at serious risk of bias in at least one domain but not at critical risk of bias in any domain.
Karas et al. [32]	ROBINS-I	Serious risk of bias	The study is judged to be at serious risk of bias in at least one domain but not at critical risk of bias in any domain.
Crawford et al. [34]	RoB 2.0	Some concerns	The study is judged to raise some concerns in at least one domain for this result but not to be at high risk of bias for any domain.
Goeb et al. [39]	ROBINS-I	Serious risk of bias	The study is judged to be at serious risk of bias in at least one domain but not at critical risk of bias in any domain.
Tang et al. [43]	ROBINS-I	Serious risk of bias	The study is judged to be at serious risk of bias in at least one domain but not at critical risk of bias in any domain.

**Table 4:** Product summary.

Device	Sensor	Price	Battery life	Tracking features
340 Sportline pedometer	Accelerometer	7.95$	12 months	Steps, distance
Kenz Lifecorder e-STEP	Accelerometer	8.06$	9 months	Steps, calories, intensity minutes
New lifestyles NL-1000	Accelerometer	54.95$	18 months	Steps, distance, intensity minutes
New lifestyles NL-800	Accelerometer	49.95$	18 months	Steps, distance
New lifestyles NL-2000i	Third-generation accelerometer, 3D (triaxial) piezoelectric resistance sensor	69.95$		Steps, calories
Letscom ID115Plus HR	Optical HR sensor, GPS KIONIX KX022-1020 sensor	30$	5 to 10 days	Steps, calories, distance, heart rate, sleep, 14 sports profile
Withings Nokia®go	Day and night sensor, motion sensor	49.95$	8 months	Steps, calories, distance, intensity minutes, sleep, swimming, running
Fitbit charge 5	Accelerometer, built-in GPS, GLONASS, optical heart-rate tracker, multipurpose electrical sensors compatible with the EDA scan app, ambient light sensor, vibration motor	149.95$	7 days	Steps, calories, distance, heart rate, sleep, SpO2
Fitbit luxe	Accelerometer, optical heart rate, ambient light sensor, vibration motor	129.95$	5 days	Steps, calories, distance, heart rate, sleep, menstrual cycle, SpO2
Fitbit inspire 2	Accelerometer, optical heart rate, vibration motor	99.95$	10 days	Steps, calories, distance, heart rate, sleep, menstrual cycle, water intake, weight
Garmin vivosmart 5	Pulse OX sensor, GPS, bike speed sensor, Garmin elevate™ heart rate technology sensor, barometric altimeter	149.99$	7 days	Steps, calories, heart rate, energy level, sleep, menstrual cycle, water intake, distance, SpO2, stress
Garmin vivofit 4	Accelerometer		12 months	Steps, calories, distance, sleep
Garmin vivosmart 4	Accelerometer, Garmin elevate wrist heart rate monitor, barometric altimeter, ambient light sensor, pulse ox		7 days	Steps, calories, distance, heart rate, sleep, floors climbed, intensity minutes, stress, gym activity profiles, swim profile
Apple watch series 3	GPS, GLONASS, Galileo, and QZSS, altimeter, optical heart sensor, accelerometer, gyroscope, ambient light sensor	199-229$	18 hours	Steps, calories, distance, activity intensity, heart rate, sleep
Apple watch series 7	GPS, GLONASS, Galileo, QZSS, and BeiDou, compass, blood oxygen sensor, third-generation optical heart sensor, accelerometer, gyroscope, ambient light sensor	399-799$	18 hours	Steps, calories, distance, activity intensity, heart rate, sleep, SpO2, ECG
Apple watch SE	GPS, GLONASS, Galileo, and QZSS, compass, altimeter, second-generation optical heart sensor, accelerometer, gyroscope, ambient light sensor	279-329$	18 hours	Steps, calories, distance, activity intensity, heart rate, sleep

## Data Availability

No additional datasets were generated or analysed during the current study.
